# Improved chromosomal-level genome assembly and re-annotation of leopard coral grouper

**DOI:** 10.1038/s41597-023-02051-z

**Published:** 2023-03-22

**Authors:** Wentao Han, Shaoxuan Wu, Hui Ding, Mingyi Wang, Mengya Wang, Zhenmin Bao, Bo Wang, Jingjie Hu

**Affiliations:** MOE Key Laboratory of Marine Genetics and Breeding, College of Marine Life Sciences/Key Laboratory of Tropical Aquatic Germplasm of Hainan Province, Sanya Oceanographic Institution, Ocean University of China, Qingdao/Sanya, China

**Keywords:** Comparative genomics, Animal breeding, Conservation genomics

## Abstract

*Plectropomus leopardus*, as known as leopard coral grouper, is a valuable marine fish that has gradually been bred artificially. To promote future conservation, molecular breeding, and comparative studies, we generated an improved high-quality chromosomal-level genome assembly of leopard coral grouper using Nanopore long-reads, Illumina short reads, and the Hi-C sequencing data. The draft genome is 849.74 Mb with 45 contigs and N50 of 35.59 Mb. Finally, a total of 846.49 Mb corresponding to 99.6% of the contig sequences was anchored to 24 pseudo-chromosomes using Hi-C technology. A final set of 25,965 genes is annotated after manual curation of the predicted gene models, and BUSCO analysis yielded a completeness score of 99.5%. This study significantly improves the utility of the grouper genome and provided a reference for the study of molecular breeding, genomics and biology in this species.

## Background & Summary

Groupers (Family Epinephelidae, Subfamily Epinephelinae) are prominent marine fishes, mostly distributed in tropical and temperate marine areas, comprising 167 species that belong to 15 genera^1^. Due to their high protein, low fat, tender meat quality, and good taste, groupers are high-quality economic fish species in Asia^2,3^. Given the huge commercial interests at stake, groupers are highly susceptible to human-induced impacts, including overfishing, making them considered threatened by the International Union for Conservation of Nature (IUCN)^4^. Therefore, how to scientifically develop and protect their resources has become the top priority^5^.

The leopard coral grouper (*Plectropomus leopardus*) has a beautiful skin color and is a valuable marine fish that commands a higher price^6–8^. Wild populations are suffering sharp declines due to overfishing and the destruction of spawning aggregations^9^. In recent years, the increasing market demands have promoted the development of artificial breeding in leopard coral grouper^10–12^. A high-quality reference genome resource has become increasingly important to facilitate the genomic breeding program, biological phenomena investigation and germplasm conservation^13,14^. Although the leopard coral grouper genome has been released^6,8,15^, the completeness of genome assembly and annotations still need to be further improved. For examples, the reported chromosomal-scale assembly of the sequence contigs only anchored 87.7% of the whole genome sequence using Hi-C technology^6^. Additionally, a wide range of gene structure annotation errors existed in the previous versions^15^, or the annotation information is not released and accessible to the public^8^.

In the present study, we generated an improved high-quality chromosome-level genome assembly of leopard coral grouper using Nanopore long-reads, Illumina short reads, and the Hi-C sequencing data. Approximately 849.74 Mb genome was assembled, consisted of 45 contigs with the contig N50 length of 35.59 Mb. A total of 846.49 Mb (99.6%) of the assembled sequences were anchored to 24 pseudo-chromosomes with low missing bases, only about 2, 354 gaps. Based on this improved genome assembly, we have significantly improved upon previous gene annotations combining *de novo* prediction, homology-based searches and transcriptome-assisted methods. BUSCO alignment showed that our final assembly contained 4, 469 (97.5%) complete BUSCOs. Taken together, this high-quality reference genome provides a valuable basis for the conservation and utilization of germplasm resources, and the further genetic breeding program in leopard coral grouper.

## Methods

### De novo genome assembly

First, we estimated the genome size and heterozygosity of leopard coral grouper using GenomeScope v2.0^16^ by *k*-*mer* analysis with clean Illumina short data. Program ontbc (https://github.com/FlyPythons/ontbc) was used to filter the Nanopore raw reads with parameters “-min_score 7 -min_length 1000”. Then, the filtered Nanopore reads self-corrected the base errors by the long-read assembler NextDenovo v2.3 (https://github.com/Nextomics/NextDenovo). Finally, clean long reads were assembled using NextDenovo v2.3 (https://github.com/Nextomics/NextDenovo) with the parameters: read_cutoff = 5k’ and ‘seed_cutoff = 40k’. We used purge_dups v1.2.5^17^ to remove the haplotypic duplication after mapping the Nanopore reads with minimap2 v2.1^18^. The assembly sequence was then polished using NextPolish v1.3.1^19^ with default parameters based on Nanopore long reads. To ensure high accuracy of the genome assembly, Illumina paired-end clean reads were aligned to the assembly using BWA v0.7.15^20^, and the results were used to conduct another round of polishing by Pilon v1.23^21^ with the parameters:--fix SNPs, indels. The contig-level assembly covered 849.74 Mb of the genome consisted of 45 contigs with a contig N50 value of 35.59 Mb.

### Hi-C analysis and chromosome assembly

**Fig. 1:**
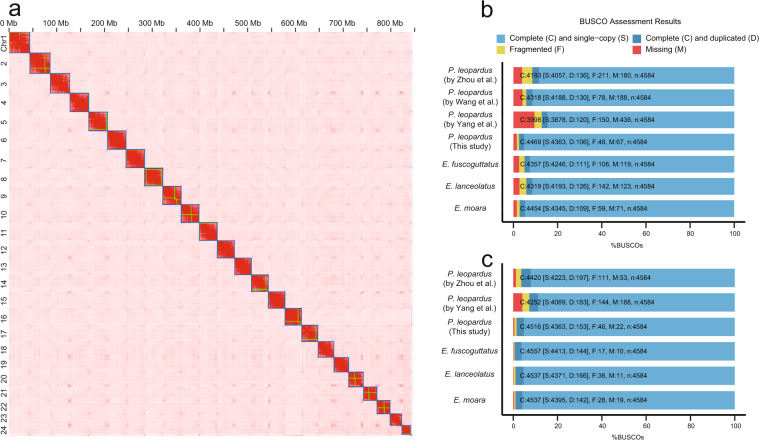
Statistics on genome assembly and Comparison of four version annotations of the leopard coral grouper, *Plectropomus leopardus*. (**a**) Hi-C interaction heat map for *Plectropomus leopardus*. (**b**) BUSCO evaluation on the genome assembly completeness. (**c**) BUSCO evaluation on the predicted gene models.

To obtain the chromosome-level genome, we further anchored all 45 contigs of the draft assembly onto 24 chromosomes using a 3D-DNA pipeline (version 201008)^22^ based on the published high-quality HiC reads^15^. The HiC reads were aligned to the polished genome using Juicer v1.5.7 software^23^ with default parameters. Mis-joins, order and orientation were corrected by the 3D-DNA pipeline^22^ with the following parameters: -r 2. After the first round of 3D-DNA, we manually adjusted the assembly with Juicebox^23^ and rerun the 3D-DNA. The Hi-C scaffolding resulted in 24 chromosome-length scaffolds (Fig. 1a).

### Repeat annotation

*De novo* and structure-based searches were used to identify repetitive sequences with both RepeatModeler v2^24^ (http://www.repeatmasker.org/RepeatModeler/) and RepeatMasker v4.0.9^25^ (http://www.repeatmasker.org). Candidate LTR-RTs repetitive sequence library was identified using LTR_finder^26^ with parameters ‘-D 15000 -d 1000 -L 7000 -l 100 -p 20 -C -M 0.9’ and LTRharvest v1.5.8^27^ with parameters ‘-minlenltr 100 -maxlenltr 7000 -mintsd 4 -maxtsd 6 -motif TGCA -motifmis 1 -similar 85 -vic 10 -seed 20 -seqids yes’. The identified LTR-RT candidates were filtered with LTR_retriever v2.5^28^ program with default parameters. RepeatScout v1.0.5^29^ LTR_retriever v2.5^28^ and RepeatModeler v2^24^ were used to build *de novo* repeat libraries. The combined repeat library was used as the final library to identify repetitive sequences using RepeatMasker v4.0.9^25^ with parameters ‘-q -no_is -norna -nolow -div 40’.

### Gene prediction and annotation

To comprehensively annotate genes, protein-coding genes prediction was undertaken using the BRAKER v2.1.5^30^ annotation pipeline which integrated different evidence, including *de novo* prediction, homology-based searches and transcriptome-assisted methods. First, for *de novo* gene prediction, we downloaded published RNA-seq (SRP201943^31^ and SRP329031^32^) and then mapped to the soft masked genome using Hi-SAT2 v. 2.1.0^33^. Then, all mapping results were used to build transcript models using BRAKER v2.1.5^30^ and StringTie v2.1.6^34^. BRAKER v2.1.5^30^ was run with Semi-HMM-based Nucleic Acid Parser (SNAP, v2013.11.29)^35^ and Augustus v3.3.3^36^ which pre-trained using released gene models of *P*. *leopardus*^6,15^. Second, protein-coding sequences of from *P*. *leopardus*^6,15^, *E*. *fuscoguttatus*^37^, *E*. *lanceolatus*^38^, and *E*. *moara*^39^ were aligned to the genome assembly using TBLASTN and GeneWise v2.2.0^40^. Third, Trinity v2.1.1^41^ was used to generate the transcripts. The transcriptome data were further assembled using the PASA pipeline v2.5.2^42^ with BLAT v35^43^ and GMAP (version 20150921)^44^ as the aligner. Finally, all evidences were merged to form a consensus gene set using EVidenceModeler v1.1.1^45^. Finally, we identified a total of 25,965 protein-coding genes (Table 2). The noncoding RNA genes including rRNAs, tRNAs, snRNAs and miRNAs were screened using INFERNAL v 1.1.2^46^ and tRNAscan-SE v1.4^47^. Four types of noncoding RNAs, including 746 miRNAs, 1,224 tRNAs, 439 rRNAs and 596 sRNAs, were identified from the *P*. *leopardus* genome (Table 3).

In order to explore the function of predicted protein-coding genes in leopard coral grouper, InterPro30, Pfam32, PANTHER 14.1, Superfamily 1.75, Gene3D 4.2.0, SMART 7.1 and TrEMBL32 databases were respectively used to predict protein function based on the conserved protein domains by InterProScan v5.36^48^. We performed functional annotation by aligning the protein sequences to NCBI nr databases and SwissProt using BLASTP. The result showed more than 99.9% (25,927) of protein-coding genes were annotated (Table 4).

## Data Records

The assembled genome has been deposited at GenBank under the accession GCA_026936395.1^49^. Moreover, the whole genome sequence data reported in this paper have been deposited in the Genome Warehouse in National Genomics Data Center^50,51^, Beijing Institute of Genomics, Chinese Academy of Sciences/China National Center for Bioinformation, under accession number GWHBPCI00000000 that is publicly accessible at https://ngdc.cncb.ac.cn/gwh/Assembly/29542/show^52^. In addition, the genome annotation files had been submitted at the figshare^53^. The Nanopore long reads, Illumina genomic sequencing data and Hi-C data were downloaded from CNGBdb^51,54^ under the accession CNP0000859^55^. Transcriptomic sequences can be retrieved under the following accession numbers: SRP201943^31^ and SRP329031^32^.

## Technical Validation

**Fig. 2:**
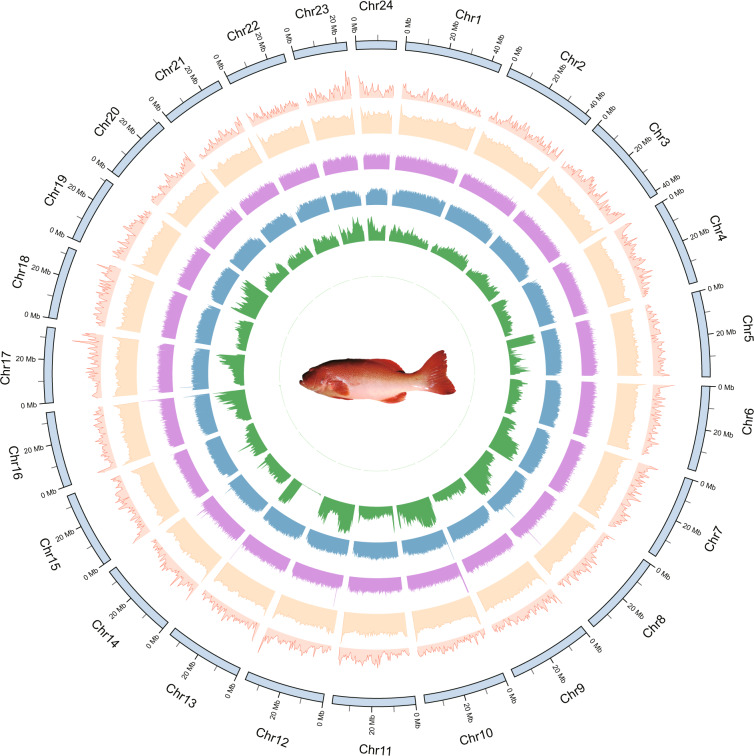
Global genome landscape of the leopard coral grouper, *Plectropomus leopardus*. From outer to inner circles: Density of genes with 500 kbp windows, ranging from 0 to 70; GC content with 500 kbp windows, ranging from 0.30 to 45; depth of coverage of Nanopore reads with 100 kbp windows, ranging from 20 to 150; depth of coverage of Illumina short reads with 100 kbp windows, ranging from 10 to 35; distribution of heterozygous SNPs with 500 kbp windows, ranging from 0 to 3,420; distribution of homozygous SNPs with 500 kbp windows, ranging from 0 to 3,420.

To evaluate the quality of genome assembly, first, we assessed genome continuity with QUAST v5.0.2^56^. Contig N50 (the length such that half of all sequence is in contigs of this size) has achieved a significant improvement to 35.59 Mb, which is much higher than other versions^6,8,15^ or closely related species (*Epinephelus fuscoguttatus*, *Epinephelus lanceolatus*, *Epinephelus moara*) assembled with long-read sequencing from 0.12 to 13.8 Mb. Meanwhile, in the latest version, there are very few gaps in the genome (2.77 per 100 kbp), which is remarkably less than the previous from 68.31 per 100 kbp to 1793.38 per 100 kbp^6,8,15^ (Table 1; Fig. 2). Second, Illumina paired-end clean reads and Nanopore long reads were mapped to the final reference genome assembly by using BWA v0.7.15^20^ and Minimap2 v2.1^18^, respectively. The mapping rate of Illumina and Nanopore reads reached 99.18% and 99.95%. We only detected 6, 900 (0.0008%) conflicting sites in the final assembly, indicating that this is a high level of the complete genome (Fig. 2; Table 5). Finally, we evaluated the completeness of our genome assembly using Benchmarking Universal Single-Copy Orthologs (BUSCO, v3.0)^57^ with the actinopterygii_odb9 database. The actinopterygii_odb9 database contained 4,584 conserved core genes while our assembled genome contained 4,469 (97.5%) of the expected actinopterygii genes (including 4,393 (95.2%) single and 106 (2.3%) duplicated ones). Obviously, our data had complete gene coverage, and 48 (1.0%) were identified as fragmented, respectively, while 67 (1.5%) were missing in our assembled genome (Fig. 1b). Furthermore, we also used BUSCO to evaluate the completeness of gene annotations^57^, and only 22 (0.5%) genes were missing in the final annotation version (Fig. 1c) Table 5.

**Table 1:** Comparison of genome assembly metrics in groupers.

	*P. leopardus*				*E*. *fuscoguttatus*	*E. lanceolatus*	*E*. *moara*
	This study	Zhou *et al*.	Wang *et al*.	Yang *et al*.			
Sequenced genome size (Mb)	849.74	881.55	913.38	787.06	1,047.01	1,087.42	1,030.48
Contig N50 (Mb)	35.59	0.86	1.41	1.14	13.80	0.12	2.22
Scaffold N50 (Mb)	38.02	34.15	40.04	33.85	44.42	46.23	43.43
Gap size (N’s per 100 kbp)	2.77	1,793.38	79.43	68.31	1.96	3,609.92	2,988.63
Complete BUSCOs (%)	97.5	91.5	94.2	87.2	95.0	94.2	97.2
Fragmented (%)	1.0	3.9	1.7	3.3	2.4	3.1	1.3
Missing (%)	1.5	4.6	4.1	9.5	2.6	2.7	1.5
Duplicate copy (%)	2.3	3.0	2.8	2.6	2.4	2.7	2.4

**Table 2:** Comparison of the genome-wide statistics for annotations of groupers.

	*P. leopardus*				*E*. *fuscoguttatus*	*E. lanceolatus*	*E*. *moara*
	This study	Zhou *et al*.	Wang *et al*.	Yang *et al*.			
Number of protein-coding genes	25,965	25,763	24,700	22,317	23,813	24,067	23,588
Average gene length (bp)	15,512	15,894	16,882	20,758	22,277	21,997	21,583
Average exon length (bp)	174	171	183	276	175	174	174
Average exon number per gene	9.2	8.4	8.7	11.2	10.5	10.3	10.4
Average intron length (bp)	1,840	1,688	1,879	1,890	2,148	2,146	2,094
Percentage of repeat sequence (%)	37.35	33.91	38.02	36.18	41.28	40.17	38.85
LTR (%)	1.69	1.35	2.68	2.12	5.18	3.68	3.45
LINE (%)	3.21	2.87	3.45	3.24	4.84	4.67	4.16
SINE (%)	0.40	0.39	2.17	0.42	0.48	0.50	0.51
DNA transposons (%)	13.58	11.35	12.80	12.79	16.60	16.82	15.87

**Table 3:** The statistics of functional annotation in the leopard coral grouper.

ncRNA type		Copy	Proportion in Genome (%)
miRNA		746	0.075
tRNA		1,224	0.011
rRNA	18 S	152	0.023
	28 S	117	0.033
	5.8 S	22	0.001
	5 S	148	0.002
	Subtotal	439	0.059
sRNA	CD-box	135	0.002
	HACA-box	80	0.001
	Splicing	380	0.006
	Subtotal	596	0.009

**Table 4:** The statistics of functional annotation in the leopard coral grouper.

Type	Number of overall predicted genes	Percentage of overall predicted genes (%)
Total	25,965	—
SwissProt	21,331	82.2
KEGG	15,813	61.0
NR	23,027	88.7
GO	15,965	61.5
Pfam	20,201	77.8
Annotated	25,927	99.9
Unannotated	38	0.1

**Table 5:** The statistics of the leopard coral grouper (MGB_pleo_1.0) SNPs.

Type	Number	Percentage (%)
All SNP	2,326,997	0.2738
Heterozygous SNP	2,320,097	0.2730
Homologous SNP	6,900	0.0008

## Data Availability

The data analyses were performed according to the manuals by the developers of corresponding bioinformatics tools and all software, and codes used in this work are publicly available, with corresponding versions indicated in Methods.
